# Are Serum Mac 2-Binding Protein Levels Elevated in Esophageal Cancer? A Control Study of Esophageal Squamous Cell Carcinoma Patients

**DOI:** 10.1155/2018/3610239

**Published:** 2018-04-17

**Authors:** Ufuk Cobanoglu, Duygu Mergan, Ahmet Cumhur Dülger, Sebahattin Celik, Ozgur Kemik, Fuat Sayir

**Affiliations:** ^1^Department of Thoracic Surgery, Medical Faculty, University of Van Yuzuncu Yil, Van, Turkey; ^2^Department of Thoracic Surgery, Training and Research Hospital, Van, Turkey; ^3^Department of Gastroenterology, Medical Faculty, University of Van Yuzuncu Yil, Van, Turkey; ^4^Department of General Surgery, Medical Faculty, University of Van Yuzuncu Yil, Van, Turkey; ^5^Department of Surgical Oncology, Medical Faculty, University of Van Yuzuncu Yil, Van, Turkey

## Abstract

**Objective:**

Elevated serum Mac 2-binding protein (M2BP) levels have been observed in some cancers. As far as we know, its importance has not been investigated in esophageal squamous cell carcinoma (ESCC). The investigated problem of this study was to evaluate whether there was a difference between ESCC patients and the control group in terms of M2BP. Also, we evaluated the diagnostic performance of serum M2BP alone or in combination with the CEA for patients with ESCC.

**Material and Methods:**

Blood serum samples were collected from 50 healthy donors and 150 patients with ESCC. M2BP levels of all 200 samples were quantified by ELISA (enzyme-linked immunosorbent assay). Patients who had been diagnosed with ESCC and did not have any other malignancies were enrolled to study.

**Results:**

The two groups did not significantly differ in terms of age (*p* > 0.05). In the control group, the mean serum M2BP level was 14.97 ± 3.46 ng/mL. The mean serum M2BP level of the ESCC patients was 176.65 ± 22.14 ng/mL. The serum M2BP level was significantly higher in patients with ESCC than in the control group (*p* < 0.001). Gender was also comparable in both groups (*p* = 0.695).

**Conclusions:**

Our analysis demonstrated that this marker may be associated with the mechanism of the disease. Despite that serum M2BP is not a specific marker for ESCC, it can be used as an adjuvant biomarker for the diagnosis of ESCC.

## 1. Introduction

Esophageal squamous cell carcinoma (ESCC) is one of the most common malignancies worldwide, being the eighth major cause of cancer-related deaths [1]. ESCC is a distinct histological type of esophageal malignances [1, 2]. This cancer is usually detected in the advanced stage of disease, when regional and/or distant metastases are already present [2, 3]. Therefore, ESCC patients usually have a poor prognosis. In ESCC, dissemination of the disease at diagnosis is favored by the lack of a serosal layer and the presence of a rich network of submucosal lymphatic vessels in the esophagus [1, 3]. Hence, ESCC patients usually have unexpected micrometastases in the tumor region at the time of diagnosis.

Currently, it is accepted that carcinogenic transformation of cells is characterized at the molecular level by, among other things, changes in protein expression [4, 5]. These proteins can provide a valuable insight into premature progression of esophageal cancer. New biomarkers and near imaging techniques may be of potential value for the diagnosis and monitoring therapy in ESCC patients. The determination of biological markers with the use of simple and noninvasive techniques to isolate them becomes important. Many researches such as ours try to determine the protein level in serum/or tumor tissues in ESCC patients by enzyme-linked immunosorbent assay (ELISA).

M2BP is known as a glycoprotein that is ~90–70 kDa. It is associated with macrophage-lectin, Mac 2, and a member of the cysteine-rich domain family of macrophage receptors [6–8]. Researches using cultured tumor cells have demonstrated that M2BP upregulates the expression of adhesion molecules [9].

Our study is aimed at investigating the potential role of this molecule as a biomarker for ESCC. For this purpose, we intended to measure M2BP levels of patients and compare these levels with control groups' levels.

## 2. Material and Methods

Blood samples were collected from 50 healthy volunteers (aged 28–70 years, median age 52.94 ± 9.87 years; 28 females and 22 males) and 150 ESCC patients (aged 28–73 years, median age 54.72 ± 9.04 years; 55 females and 95 males) at initial disease diagnosis before tumor resection at the Yuzuncu Yil University General Surgery, Thoracic Surgery, Gastroenterology Department, in Van. The patient and control group characteristics are described in Table 1. Patients did not undergo any treatment (surgery, radiotherapy, or chemotherapy) before peripheral blood samples were collected. Patients who had been diagnosed with ESCC and did not have any other malignancies were enrolled to study. All patients were informed about the study, and informed consent was taken.

### 2.1. Collection of Serum Samples

2 mL of collected peripheral blood was quickly transferred to a tube and allowed to settle at room temperature (23–25°C) for 15–20 min. 1 mL of supernatant was aspirated and transferred to a clean 1 mL centrifuge tube, and the following steps were completed within 1 h or 2 h (at 4°C): centrifugation at 1000 rpm for 15 min. at 4°C, collection of supernatant, and storage at −80°C for future use.

### 2.2. Biochemical Analysis

Serum levels were analyzed in duplicate with inclusion of two quality control samples in every run. The M2BP level was measured with a commercially available sandwich ELISA (Human Mac-2BP ELISA kit, code: ab119502, Abcam) test. The test's sensitivity was 0.92 ng/mL (range: 12.5–200 ng/mL). CEA was measured using a commercially available automated ELISA kit (CEA Immunoassay, Roche Diagnostics, USA). The upper limit of normal for CEA was 5 ng/mL.

### 2.3. Statistical Analysis

Shapiro-Wilk normality test, Q-Q plot, histogram, and box plot graphs were drawn. Descriptive statistics included mean, standard deviation, median, minimum, maximum, frequency, and percentage. The independent samples *t*-test was used for normally distributed continuous variables and the Mann–Whitney *U* test for nonnormally distributed continuous variables. Nominal variables were analyzed with the chi-square test with Yates correction. A double-sided *p* value of <0.05 was considered statistically significant. Statistical analyses were performed using SPSS 21 software.

## 3. Results

Table 1 provides demographic and clinicopathological data as well as the results of the whole study population. The serum M2BP level was measured in 50 healthy subjects (22 males and 28 female) with a mean age of 52.94 ± 9.87 years (range 28–70 years). The study group included 150 patients with ESCC (95 males and 55 females) with a mean age of 54.72 ± 9.04 years (range 28–73 years). Equal variances assumed in the age were 0.239.

**Figure 1:**
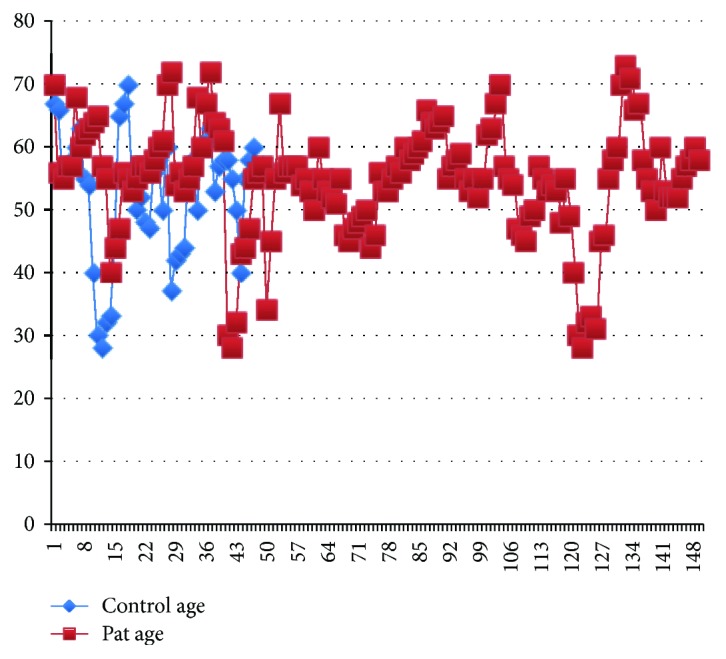
The histogram of the ages of all samples (pat = patients; cont = control group).

**Figure 2:**
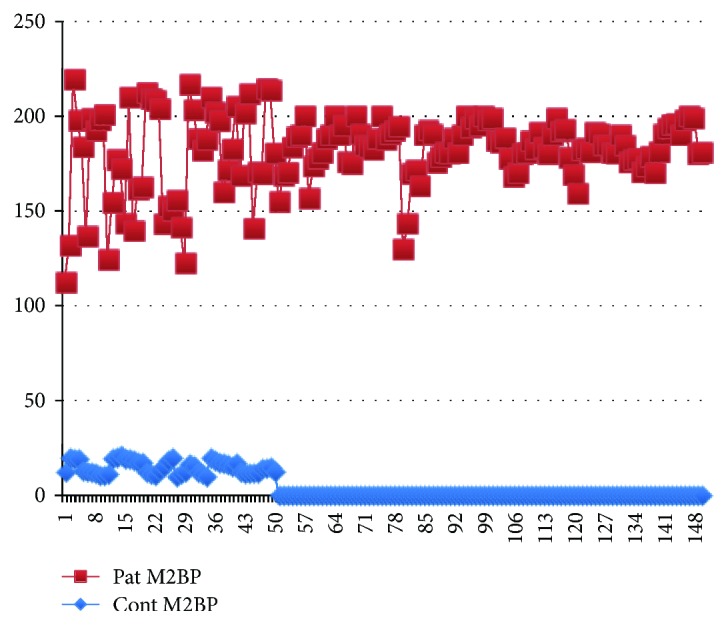
The histogram of the M2BP levels of the control group and all patients.

The two groups did not significantly differ in terms of age (*p* > 0.05) (Figure 1). In the control group, the mean serum M2BP level was 14.97 ± 3.46 ng/mL. The mean serum M2BP level of the ESCC patients was 176.65 ± 22.14 ng/mL. The serum M2BP level was significantly higher in patients with ESCC than in the control group (*p* < 0.001) (Figure 2). Age was similar in both groups (*p* = 0.621). Gender was also comparable in both groups (*p* = 0.695).

## 4. Discussion

In our study, we found an elevated serum M2BP concentration in ESCC patients. The serum M2BP level was significantly increased in patients with ESCC than in healthy controls. CEA alone has a poor sensitivity for the diagnosis of ESCC. Some researchers have reported that combinations of tumor markers can considerably increase diagnostic efficiency than single markers can [10]. Our results showed that, in light of its poor sensitivity, CEA falls short of the identification of ESCC. In contrast, M2BP had an excellent sensitivity for the identification of ESCC. Furthermore, the M2BP concentration was elevated in ESCC, which can differentiate ESCC patients from healthy individuals with a lower margin of error. M2BP was investigated for both lung and prostate cancer and some other cancers [11–13]. This protein has been demonstrated to be a ~90–70 kDa glycoprotein, which might be a part of galectin-3 [14]. Studies employing cDNA cloning indicated that it comprises a cluster of cysteine and *N*-glycosylation sites and that its N-terminal components share a similar structure with the extracellular domain of the macrophage scavenger receptor [8, 14]. It was reported that M2BP has a role in galectin-1 function and modulates cell aggregation by this lectin [15]. Those mentioned properties made us conclude that M2BP has a function in cancer cell metastatic activity, and another explanation for M2BP is that M2BP plays a role in the immune system [16]. Another opinion is about the role of M2BP in cellular immune reactions. Researches claimed that M2BP stimulates monocytes and increases IL-2 levels [8, 17]. And by doing these, it increases intercellular adhesion molecule-1 (ICAM-1) and vascular cell adhesion molecule-1 (VCAM-1) in endothelial cells of tumor [18]. M2BP may be responsible for an elevated immune reaction against tumor cells. Some chemotherapy agents, like those agents used in ESCC, exhibit their toxic effect on tumor cells via increasing apoptosis [19, 20]. Although apoptosis in cancer cells has not been well understood, there are some studies claiming that resistance to chemotherapy-induced cancer cell death might be due to some matrix proteins [21, 22]. As a matrix protein, M2BP, which seems to be mediated by *β*1 integrins and is independent of galectin-3, may play a strong role in apoptosis of cancer cells by means of cellular interactions [23].

## 5. Conclusions

Our study showed that M2BP concentration was significantly elevated in serum in ESCC patients than in the control group. In accordance with our results, we propose that M2BP could be used as a biomarker in early ESCC and may also be a prognostic marker for ESCC. For early diagnosis and for improving patients' survival, we need some kind of markers which could be specific and easily applicable. These biomarkers should be noninvasive, sensitive, and used by a simple method.

## Figures and Tables

**Table 1:** The demographic variables and M2BP levels of both the control group and patients.

	Controls (*n* = 50)	Patients (*n* = 150)	*p*
Age years	52.94 ± 9.87	54.72 ± 3.46	>0.05
Min–max	(28–70)	(28–73)	
Gender (M/F)	(22/28)	(55/95)	
Total M2BP (ng/mL)	14.97 ± 3.46	176.65 ± 22.14	<0.001
Min–max	(10–21.3)	(40.2–192.5)	
Percentile 50 median	182.400		
CEA (ng/mL)		4.26 ± 1.37	
Min–max		(2–5)	
Percentile 50 median	4.800		
